# Alzheimer’s Disease: A Brief History of Immunotherapies Targeting Amyloid β

**DOI:** 10.3390/ijms24043895

**Published:** 2023-02-15

**Authors:** Anne-Cathrine S. Vogt, Gary T. Jennings, Mona O. Mohsen, Monique Vogel, Martin F. Bachmann

**Affiliations:** 1 Department of Rheumatology and Immunology (RI), University Hospital, 3010 Bern, Switzerland; 2 Department for BioMedical Research (DBMR), Faculty of Medicine, University of Bern, 3008 Bern, Switzerland; 3 Graduate School for Cellular and Biomedical Sciences (GCB), University of Bern, 3008 Bern, Switzerland; 4 Saiba AG, 8808 Pfäffikon, Switzerland; 5 Centre for Cellular and Molecular Physiology (CCMP), Nuffield Department of Medicine, The Jenner Institute, University of Oxford, Oxford OX3 7BN, UK

**Keywords:** Alzheimer’s disease, amyloid-β, immunotherapy

## Abstract

Alzheimer’s disease (AD) is the most common form of dementia and may contribute to 60–70% of cases. Worldwide, around 50 million people suffer from dementia and the prediction is that the number will more than triple by 2050, as the population ages. Extracellular protein aggregation and plaque deposition as well as accumulation of intracellular neurofibrillary tangles, all leading to neurodegeneration, are the hallmarks of brains with Alzheimer’s disease. Therapeutic strategies including active and passive immunizations have been widely explored in the last two decades. Several compounds have shown promising results in many AD animal models. To date, only symptomatic treatments are available and because of the alarming epidemiological data, novel therapeutic strategies to prevent, mitigate, or delay the onset of AD are required. In this mini-review, we focus on our understanding of AD pathobiology and discuss current active and passive immunomodulating therapies targeting amyloid-β protein.

## 1. Introduction

Most proteins need to fold into specific three-dimensional structures to become functional [1]. The aberrant folding of proteins has been linked to a rapidly expanding list of pathologies [1]. A group of diseases occurs due to toxic gain of function mutations, where metastable proteins undergo aggregation leading to intra- or extracellular deposits of toxic insoluble fibrillar proteins [1,2,3]. Today there are around 30 human diseases associated with protein misfolding and amyloid formation [4]. These pathologies include neurodegenerative disorders such as Alzheimer’s disease (AD) and Parkinson’s disease (PD) as well as type II diabetes (T2DM) [1].

Alzheimer’s disease (AD) is one of the most common irreversible, neurodegenerative disorders worldwide [5,6]. AD is the most common cause of dementia, affecting 50 million people with cases rising to approximately 150 million by 2050 [6]. Usually, the disease affects people at age 60 and above [7]; however, the accumulation of Aβ plaques usually starts 10–20 years before clinical manifestation [8]. The neuropathology of AD is defined by the accumulation of extracellular plaques containing amyloid β protein as well as the increase in intracellular tau-containing neurofibrillary tangles [9]. These specific hallmarks trigger neuronal dysfunction, neurotoxicity, and inflammation, leading to cognitive dysfunction, and affecting memory and behavior [10]. During the primary stage of the disease, para-hippocampal brain regions, which are responsible for the formation of new memories in the brain, are affected by neuronal and synaptic impairment. With the progression of the disease, neuropathology continues to spread, causing a total brain mass reduction of up to 35% [11]. Through continuous spreading, patients suffer from progressive disability due to neuronal loss and cognitive decline throughout the disease course, with death occurring within 5–12 years of symptom onset [12]. With the rise of AD cases, the costs of care associated with the disease are increasing [13]. The massive impact on caregivers as well as the public health system is staggering [14]. In terms of total costs to society, AD is the third most costly disease in the world after cancer and coronary heart disease. The total economic cost of AD in 2020 is estimated to be USD 305 billion [15]. Total costs include direct, indirect, and intangible costs. Direct costs include different medical care costs (e.g., nursing home care, medications, physician visits, hospitalizations) and nonmedical care costs (e.g., home health helps, private social care, adult day care). Indirect costs are imputed values of resources lost due to the illness, including premature deaths, patient and caregiver lost productivity, and unpaid caregiving time [16]. Direct medical costs are shared between families and public health systems, but indirect costs fall entirely on families resulting in a high private economic burden. Intangible costs are those related to pain and suffering endured by patients and families and those related to the deterioration of patient and caregiver quality of life. The presence of comorbid conditions significantly increases the cost of caring for patients with AD. The effects of comorbidities are particularly important in AD patients as most of them have at least one comorbid condition [17]. Therefore, much effort is needed to develop therapeutic measurements for AD. So far, several disease-modifying therapies (active and passive immunization strategies), are in clinical trials, targeting amyloid-β [18].

This brief review discusses our understanding of the pathophysiology of AD and summarizes current treatment strategies targeting amyloid-β, highlighting some risk factors involved in disease development.

## 2. Disease Mechanism and Pathogenesis

### 2.1. The Amyloid Pathway

**Figure 1:**
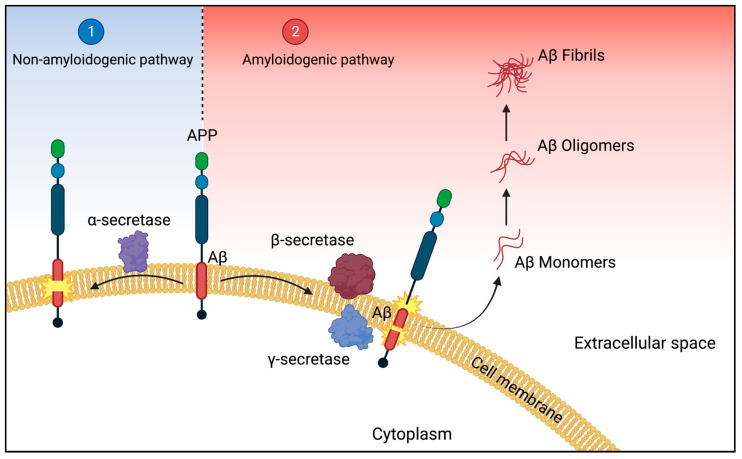
Processing of APP. (1) During the non-amyloidogenic pathway, amyloid precursor protein (APP) is cleaved by α-secretase yielding extracellular released soluble APP (left). (2) For the amyloidogenic pathway, APP is primarily cleaved by β-secretase and subsequently cleaved by γ-secretase within the membrane (right). The proteolytic processing of APP via the amyloidogenic pathway releases amyloid-β into the extracellular space, which is prone to self-aggregate, leading to the formation of cytotoxic oligomers and insoluble Aβ fibrils. Adapted from Patterson et al. [25]. This illustration was created using BioRender.com.

According to the amyloid hypothesis, synaptic dysfunction and neurodegeneration are caused by the accumulation of amyloid-β in Alzheimer’s disease (AD) [19]. The precursor of the main constituent of amyloid plaques, Aβ peptides, is the transmembrane amyloid precursor protein (APP) [20]. The APP gene is located on chromosome 21 in humans and in the brain, it is involved in the development of neurons, the formation, and repair of synapses, and the provision of synaptic plasticity [21,22]. Proteolytic sequential cleavage of the amyloid protein from APP by APP secretase is a key event in AD pathogenesis. There are two processing pathways for APP, non-amyloidogenic and amyloidogenic pathway (Figure 1): (1) α-secretase cleaves APP within the amyloid sequence either in the middle, generating soluble APPα or shorter Aβ species when further cleaved by β-secretase [22,23]. This pathway is referred to as the so-called non-amyloidogenic pathway (Figure 1). In contrast, in the second pathway (2) the sequential cleavage by β- and γ-secretase on the N- and C-terminal ends respectively, leading to the formation of soluble Aβ peptides (monomers) [24]. Soluble Aβ undergoes conformational changes allowing inter-molecule hydrogen bonding resulting in highly stable β-sheet structures, leading to pathologic aggregates in the brain, causing brain dysfunction [25] and neurodegeneration [11]. The formation of pathophysiological Aβ by the second pathway is called the amyloidogenic pathway (Figure 1) [25]. Beta-Site APP Cleaving Enzyme 1 (BACE1) is the β-secretase enzyme cleaving the extracellular region of APP, releasing the soluble N-terminus while the C-terminus remains bound to the membrane [26]. Increased β-secretase activity is related to two mutations in BACE1 (the Swedish mutation and the Italian variant) [26,27]. High BACE1 activity has been detected in human AD brain extracts [28]. The membrane-bound C-terminal fragment is further cleaved by γ-secretase, releasing Aβ-proteins at the luminal sides [29]. The catalytic units of the γ-secretase complex are presenilins 1 β and 2 (PSEN1/PSEN2) [26]. Mutations in PSEN1/PSEN2 have been related to increasing γ-secretase activity [25]. Among consecutive cleavage of APP by y-secretase at variable sites, different Aβ peptides, ranging in length from 38 to 43 residues, are produced [30]. Along Aβ species, Aβ42 and Aβ43 have the most self-aggregating potential, whereas Aβ40 is comparably more benign by even protecting aggregation of Aβ42 [31]. Aβ42 and Aβ40 are the most abundant Aβ species in the human brain and a critical biochemical feature in AD [30]. After their generation, Aβ peptides are found in different aggregation states including, monomers, oligomers, and protofibrils forming fibrils, that finally accumulate in plaques [30]. The main mediators of cytotoxicity in AD are likely to be soluble oligomeric forms of Aβ, which may multiply through a “prion-like” mechanism [32].

### 2.2. Pathogenesis and Clinical Stages

The neuropathology of the disease is characterized by the accumulation of extracellular amyloid β (Aβ) as amyloid-containing plaques and the accumulation of aggregated, phosphorylated intracellular tau protein [33]. The oligomeric forms of aggregated Aβ and tau proteins are suggested to be the most toxic species [34]. The exact structure and toxicity and ability to replicate the different Aβ-species is an area of intensive investigation. These oligomers are thought to be the primary pathogenic driver and the downstream leads to tau phosphorylation, NFT (neurofibrillary tangle) formation, and eventually synaptic and neuronal loss accompanied by neuroinflammation [35]. The deposition of Aβ starts in the basal, temporal neocortex regions of the brain, with continuous deposit formation in the hippocampus, amygdala, diencephalon, and basal ganglia [36]. This deposition and the resulting neuronal loss result in clinical symptoms such as progressive memory decline, impaired executive function, and advanced stages of the disease to severe memory loss and disorientation, and eventually death [37].

Four stages can be used to categorize the clinical stages of AD. Mild memory loss and early degenerative changes in the hippocampus and cortex characterize the first pre-clinical stage, in which daily activities are not hindered and patients do not exhibit clinical indications of AD [38]. This stage is followed by mild AD, the second stage. At this stage, patients start to show clinical symptoms, such as subtle memory deficits, loss of concentration, and disorientation of place and time [39,40]. With the spread of the disease to all areas of the cerebral cortex, memory loss increases. Individuals show impairment in speaking, writing, and reading when reaching this third stage, defined as moderate AD [41]. Severe AD, the fourth stage, involves continuous spreading and deposition of Aβ to the entire cortex area, with a severe accumulation of neuritic plaques and neurofibrillary tangles (NFT), resulting in progressive functional and cognitive impairment and eventually death [42].

## 3. Current Treatment Strategies Targeting Aβ

The misfolding and aggregation of the Aβ protein is a hallmark of AD [43]. Therefore, it is the primary goal of Aβ-immunotherapy to reduce the formation, spread, and deposition of Aβ aggregates in the human brain. It appears that the pathology of the disease begins to develop 10–20 years before the currently recognizable clinical signs of AD [35]. Therefore, novel therapeutics should aim to delay or prevent the disease at the preclinical stage. The two most comprehensive anti-Aβ therapy concepts are active as well as passive immunization [19]. Passive vaccination introduces exogenous monoclonal antibodies (mAbs) directly, whereas active vaccination uses an exogenous substance to direct the immune system to produce an immunological response [44]. The advantage of active immunotherapy is the long-term production of antibodies through short-term drug administration at a limited cost. On the other hand, the immune response can be inconsistent or absent, especially in older people [45]. In contrast to active vaccination, passive immunotherapy has the advantage of ensuring constant antibody titers and allowing control of adverse effects by discontinuing treatment. The major disadvantages of monoclonal antibodies are the need for repeated administration and the associated production costs [46]. Additionally, passive immunotherapy can cause the innate and adaptive immune systems to become overactive, which can have serious implications including cerebral vasculitis [18].

Agents that are presently undergoing clinical trials were grouped into four groups for the 2022 Alzheimer Drug Development Pipeline: disease-modifying biologics, disease-modifying small molecules, treatments against neuropsychiatric symptoms, and cognitive enhancers [47]. According to this report, 31 agents are currently in phase III clinical trials, where 5 of them fall into the category of disease-modifying agents, targeting Aβ (Table 1) [47]. Until now, anti-Aβ agents have been acting by primarily lowering Aβ production, inhibiting Aβ aggregation, and accelerating Aβ clearance [18].

### 3.1. Passive Immunotherapy Phase III Clinical Agents

There have been several clinical trials for active as well as passive immunotherapeutic interventions to treat AD, but many of these trials were terminated due to a lack of efficacy [49]. Two major obstacles were faced regarding passive immunotherapy. The blood-brain barrier (BBB) limits antibodies’ ability to enter the brain [50]. Therefore, the first challenge is to find the optimal dosage of the antibody, to cross the BBB, and to reach the area with the neuropathology [51]. The second difficulty is to avoid unforeseen neuroinflammation in the brain. There have been several clinical trials of AD agents, where brain inflammation occurred due to overactivated microglia [52]. Nevertheless, a new generation of anti-Aβ monoclonal antibodies (mAbs), has been demonstrated to prevent the formation of fibrillar aggregates from Aβ monomers, in vitro and in vivo [53]. Indeed, several anti-Aβ mAbs were very successful in decreasing brain pathology in AD animal models as well as in human patients [54].

**Figure 2:**
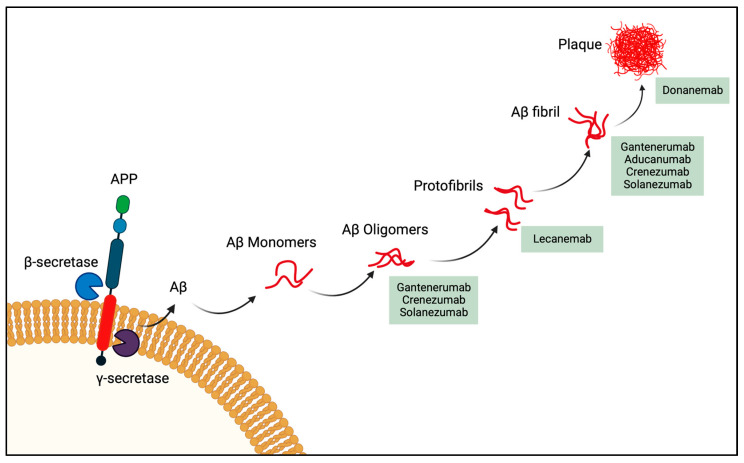
Targets of monoclonal anti-Aβ agents. The main mode of action of anti-amyloid β drugs is currently in phase III clinical trials. Adapted from Panza et al. [76]. This illustration was created using BioRender.com.

One of them is Aducanumab, a human IgG1 monoclonal antibody directing to the N-terminus of the Aβ protein (3-7) preferably targeting Aβ aggregates (Figure 2) [49,55], had a clear therapeutic effect and successfully removed Aβ from the brain of transgenic mice [49]. In June 2021 the US Food and Drug Administration (FDA)-approved Aducanumab (Aduhelm; Biogen Inc, Cambridge, MA, USA), for treating patients diagnosed with mild to moderate AD [55]. Even though this mAb significantly decreases Aβ plaques, the benefit on cognition is controversially discussed [56]. A second mAb also targeting Aβ is Gantenerumab (MorphoSys, Planegg, Germany and Hoffmann-La Roche, Basel, Switzerland). It is a human IgG1 antibody, targeting Aβ fibrils (Figure 2), and it comprises both N-terminal and central amino acids of Aβ [57] In a recent press release, Roche declared, that the phase III Graduate studies with their mAb failed the primary endpoints of slowing clinical decline in patients with early, prodromal to mild AD [58]. Additionally for Crenezumab (AC Immune SA, Lausanne, Switzerland; Genentech, South San Francisco, CA, USA; Hoffmann-La Roche), a humanized IgG4 targeting Aβ oligomers as well as fibrils [59], the clinical phase III placebo-controlled study was not successful in reducing the clinical decline in participants with early AD [60]. Solanezumab (Eli Lilly & Co., Indianapolis, IN, USA), a humanized form of the murine monoclonal antibody m266 generated against Aβ13-28 has similar binding qualities as Crezenumab [61] and finalized its phase III trial in 2014 for mild to moderate AD. However, the results were not convincing as the antibody failed to prevent a decline in cognition and functional ability [62]. Donanemab, another humanized mAb from Eli Lilly & Co., recognizing Aβ(p3-42) a pyroglutamate form of Aβ that is aggregated in amyloid plaques [63,64,65]. This humanized IgG1 antibody is currently being tested on AD patients with prodromal to moderate AD in a phase III clinical trial [66,67].

In late September 2022, Eisai and Biogen revealed promising results with their mAb Lecanemab in a phase III clinical trial [68]. Lecanemab is a humanized monoclonal IgG1 of the mouse mAb158, which selectively binds to large soluble Aβ protofibrils (Figure 2) [69,70]. This antibody was able to abolish Aβ accumulation in astrocytes and protected cultured neurons from Aβ toxicity [71]. Already in 2021, this antibody showed encouraging results in a phase IIb double-blind placebo study, where brain amyloid accumulation and disease progressions were diminished in a dose-dependent manner, in patients diagnosed with mild to moderate AD [72]. In their phase III double blind study, Lecanemab therapy reduced early AD amyloid indicators and led to a moderately less decline on measures of cognition and function [73]. It seems that Lecanemab is the most promising mAb among the five agents currently in phase III trials and which has just been registered in the United States [74]. Aducanumab and in particular, Lecanemab, showed a positive therapeutic effect on the decline in brain Aβ levels along with the slowing of cognitive decline [54,68]. However, there was a slight increase in brain bleeds in the antibody-treated group (0.6% compared with 0.2% in the placebo group) [73]. Nevertheless, it remains unclear if this is linked with blood thinners, which may increase the risk of microhemorrhage in antibody-treated Alzheimer’s patients [75].

### 3.2. Active Immunotherapy

Due to the inferior induction of cellular and humoral responses to novel antigens, especially if the response is T-cell dependent, aging-related loss in immunological functioning is the primary cause of older people’s decreased protective responses to vaccines [77]. The need for vaccine strategies to address this issue is growing, especially for active immunotherapy against age-related diseases such as AD [77].

**Figure 3:**
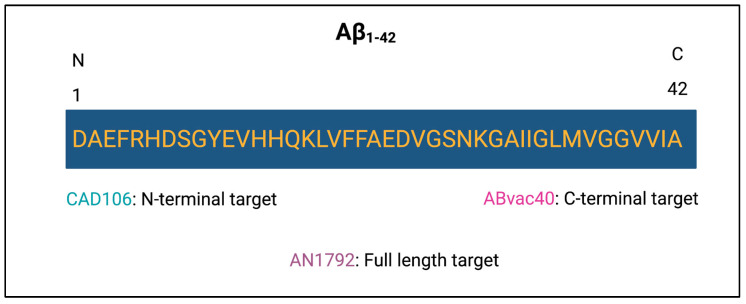
Active immunotherapy agents targeting Aβ. Adapted from Song et al. [18]. This illustration was created using BioRender.com.

The first version of a vaccine against Aβ consisted of Aβ1-42 (Figure 3), formulated with QS-21 adjuvant, called AN1792 [78]. However, clinical trials involving vaccination with Aβ1-42 had to be stopped because of the development of aseptic meningoencephalitis in 6% of the treated patients [79] due to the use of full-length Aβ which was associated with an induction of Aβ-specific T-cell mediated pro-inflammatory responses. Consequently, second-generation Aβ-active immunotherapies targeting different Aβ epitopes have been developed to control for inflammatory effects [80,81]. These novel peptide vaccines were created lacking components necessary for Aβ-specific T-cell activation, leaving only the components required to produce specific anti-Aβ antibodies [82]. One such vaccine is Novartis’ CAD106 (Amilomotide). It consists of multiple copies of Aβ1-6 (Figure 3) coupled to a virus-like particle (VLP) derived from the bacteriophage Qβ [81,83]. Transgenic mice overexpressing human amyloid precursor protein (APP) with a mutation associated with familial Alzheimer’s disease spontaneously develop plaque deposition and signs of Alzheimer`s disease. In such transgenic mice, CAD106 has been shown to interfere with Aβ aggregation and reduce the plaque burden. Phases I/II clinical trials (NCT01097096) showed a favorable safety profile and an acceptable antibody response with preliminary evidence of an amyloid reduction in patients with mild to moderate AD [84,85]. As the first active immunotherapy agent entering phase II/III (NCT0256551), CAD106 showed beneficial effects in slowing down amyloid deposition in humans [86]. For the prevention of amyloid deposition in high-risk groups, CAD106 treatment may offer a favorable risk/benefit profile at this stage [86].

Additional vaccine candidates consist of linking other B cell epitopes (Aβ1-12, Aβ33-40, Aβ1-15) either conjugated to some carrier proteins (keyhole limpet cyanine (KHL)) or with some liposome adjuvant [87]. Such a vaccine is under investigation, ABvac40 (Araclon Biotech) and targets the C-terminus of Aβ1-40 (Figure 3). It is made up of C-terminal ends of Aβ40 that have been conjugated to KHL and repeated many times to elicit an immunological response [88]. Another approach has been explored by fusing B cell epitopes (Aβ1-12) with T cell epitopes derived from tetanus toxoid to stimulate memory T cells generated by tetanus vaccination [78]. Although these latter vaccines have reached different stages of clinical development, no results have been published or have shown clear benefits [87]. In consequence, active immunotherapy requires two major adjustments: (1) vaccinations must be modified for older individuals, maybe by adding novel structural elements to elicit a potent immune response; and (2) patients with preclinical AD must be enlisted, where PET imaging and CSF biomarkers will be essential to enabling early diagnosis and tracking therapy progress in this situation [89]. Moving from a late therapeutic intervention to a prophylactic treatment. Emphasizing that a vaccine should aim to avoid premature neurodegeneration rather than to revive dying neurons [90].

## 4. Alzheimer’s Disease Risk Factors

Although Aβ is thought to be one of the main mediators of AD-related synaptic loss and eventually neuronal death, AD neuropathology involves a number of additional risk elements, which have been associated to increase the risk of developing the disease [91]:

(1) The most important risk factor for developing the disease is aging. Many epidemiological studies have shown that aging is the predominant factor for cognitive decline [92]. The age at which the first symptoms appeared can be used to classify the disease. People under 65 are affected by early-onset AD, whereas people 65 and above are affected by late-onset AD [93].

(2) The second most common risk factor is genetics [94]; 70% of the risk of developing the disease can be attributed to genetic factors [94]. Most cases of early-onset AD are inherited in an autosomal-dominant pattern due to mutations occurring in genes such as APP, PSEN1/PSEN2, and apolipoprotein E (ApoE) [95]. A total of 25 mutations have been identified on the APP gene, related to AD and causing the accumulation of Aβ [96]. While there are more than 200 mutations associated with AD in the PSEN1 gene, the PSEN2 gene has fewer variants with less than 40 mutations [96]. PSEN1 mutations often involve a single amino acid change, while severe mutations can be caused by two amino acid changes [97]. These mutations in the PSEN1 gene increase the ratio of Aβ42/Aβ40, mostly by lowering the amounts of Aβ40 produced [97]. PSEN2 mutations, in contrast, are sporadic and have a minimal impact on the generation of Aβ, but still are associated with AD [96]. For ApoE, a glycoprotein highly expressed in astrocytes, there are three different alleles, giving rise to the apoE2, apoE3, and apoE4 isoforms [98]. It has been shown that apoE4 is crucial for the formation of Aβ as senile plaque and the main risk for late-onset AD [98].

(3) Numerous neurological disorders and diseases, such as multiple sclerosis, schizophrenia, depression, and autism, are largely influenced by the developmental and physiological variations between men and women [99]. For AD, both men and women can be affected; however, women make up roughly two-thirds of AD cases. It appears that women have a higher incidence of AD in old age and have a more robust progression of moderate cognitive impairment as well as a higher severity of clinical dementia [91]. In addition, some genetic variations, such as the ApoE4 allele, more profoundly raise the risk of AD in women relative to men [100]. This issue has been investigated by Buckley et al. in 2018, where they examined the relationship between sex and cognitive impairment in relation to Aβ burden and ApoE genotype. [101]. The incidence of ApoE4 and the load of Aβ did not differ by sex; however, females with greater Aβ burden showed a faster cognitive reduction than males [101].

(4) There are some acquired factors that increase the risk of developing AD. According to Li et al. (2015), the increased risk of acquiring AD and type 2 diabetes mellitus (TDM2) is clearly linked [102]. Animal studies have shown that insulin insufficiency or resistance can stimulate the activation of β- and γ-secretase in addition to causing a reduction in Aβ clearance, resulting in its accumulation in brain tissue [98].

(5) Besides aging, genetics, and sex differences, the chance of developing AD may be increased by environmental risk factors such as food, metals, infections, and air pollution, which may cause oxidative stress and inflammation [100].

## 5. Summary and Future Perspectives

AD is a progressive neurodegenerative disease affecting people worldwide [36]. As the world population is getting older, the number of seniors, and consequently those with dementia, is climbing [103]. As its prevalence strongly increases with age, AD is the main cause of dementia in older people. When people are 65 to 74 years old, the incidence is about 3%; when people are 75 to 84 years old, it is 19%; and when people are over 84 years old, it is 47% [104]. It is now generally accepted that the pathology of the disease starts years, if not decades, before clinical manifestations become visible.

Over the past 10–15 years, the field of AD immunotherapy has grown enormously, with 143 drugs currently in the AD drug development pipeline [47]. Although many preclinical studies have reported the clearing effects of Aβ deposits by passive as well as active immunotherapy, they could not delay the progression of AD [46,69,105]. This shows that once extensive neuronal damage has taken place, eliminating amyloid aggregates will not be able to reverse cognitive deficiencies [46]. The goal of innovative therapeutic approaches is to start Aβ immunotherapy before the onset of AD pathological changes or in the very early stages of clinical symptoms. Therefore, it is urgent to change the diagnostic toward the detection of pre-symptomatic AD in order to increase our understanding of the condition and to develop better techniques for early diagnosis when neuroprotection is feasible.

Preventing dementia onset across different populations around the world is one of the global public health priorities. The estimated annual medical care associated with dementia is higher for Hispanic and Black individuals than for their White counterparts [106]. Moreover, when looking on a gender basis, the prevalence is higher in women than in men across the world and the ratio of cases in women versus men is 2:1 [15]. Alzheimer’s disease is the fourth cause of disability in women aged 60 years or older worldwide. Thus, early effective treatment can help to increase the quality of life of women and men by minimizing the risk for future dementia and thereby reducing gender inequality in AD. Alzheimer’s disease is more present in low-income and middle-income countries (LMICs) than in high-income countries, because of greater risk factor burdens such as less education, high blood pressure, obesity, physical inactivity, smoking, early life malnutrition, and survival with more infections [103]. Preventive interventions would also allow great reductions in cases of dementia in LMICs.

Many risk factors have been associated with AD including aging, genetic factors, sex differences as well as environmental components [92]. Even though the amyloid cascade holds that deposition of Aβ drives neuronal dysfunction and eventually neuronal death [94], the studies of such risk factors are important to understand the pathophysiological processes of the disease.

## 6. Conclusions

AD is an irreversible neurodegenerative disorder, which is considered a leading world health concern [100]. Even though several immunotherapeutic approaches showed promising preclinical results in animal models, the compounds then often failed in clinical trials, as they did not show beneficial effects in treating nor slowing down the progression of the disease [107]. However, hope is on the rise, since the FDA-approved Aducanumab in 2022 [55] and now Lecanemab (Leqembi^®^) via the Accelerated Approval Pathway, by the 6th of January 2023 [74]. The antibody lowered early-stage amyloid indicators and mildly decreased declines in cognition and function during cognitive assessments [73]. Instead of merely treating disease symptoms, this treatment option is the most recent medication to target and influence the underlying disease progression of AD, which could improve the lives of millions who suffer from the disease.

## Figures and Tables

**Table 1:** mAbs targeting Aβ with completed phase III clinical trials. Adapted from Pardrige [48].

Target	Drug	Sponsor	Status of Clinical Trial	ClinicalTrials.gov Identifier
Aβ aggregates	Aducanumab	Biogen	Phase III	NCT04241068
Aβ fibrils	Gantenerumab	Roche	Phase III	NCT03443973
Aβ aggregates	Crenezumab	Genentech	Phase III	NCT03114657
Amyloid β	Solanezumab	Eli Lilly	Phase II/III	NCT01900665
Aβ aggregates	Donanemab	Eli Lilly	Phase III	NCT04437511
Aβ Protofibrils	Lecanemab	Eisai	Phase III	NCT04468659

## Abbreviations

Aβ	Amyloid-β protein
AD	Alzheimer’s disease
APP	Amyloid precursor protein
mAb	Monoclonal antibody
VLP	Virus-like particle
LMIC	Low and middle-income countries
NFT	Neurofibrillary tangles

## References

[1] Hartl F.U. (2017). Protein Misfolding Diseases. Annu. Rev. Biochem..

[2] Terio K.A., O’Brien T., Lamberski N., Famula T.R., Munson L. (2004). Amyloidosis in Black-Footed Cats (*Felis nigripes*). Vet. Pathol..

[3] Westermark P., Lundmark K., Westermark G.T. (2009). Fibrils from Designed Non-Amyloid-Related Synthetic Peptides Induce AA-Amyloidosis during Inflammation in an Animal Model. PLoS ONE.

[4] Tjernberg L.O., Rising A., Johansson J., Jaudzems K., Westermark P. (2016). Transmissible Amyloid. J. Intern. Med..

[5] Galzitskaya O.V., Galushko E.I., Selivanova O.M. (2018). Studies of the Process of Amyloid Formation by Aβ Peptide. Biochemistry.

[6] Fish P.V., Steadman D., Bayle E.D., Whiting P. (2019). New Approaches for the Treatment of Alzheimer’s Disease. Bioorg. Med. Chem. Lett..

[7] Chartier-Harlin M.-C., Crawford F., Houlden H., Warren A., HUghes D., Fidani L., Goate A., Rossor M., Roques P., Hardy J. (1991). Early-Onset Alzheimer’s Disease Caused by Mutations at Codon 717 of the /J-Amyloid Precursor Protein Gene. Nature.

[8] Chapleau M., Iaccarino L., Soleimani-Meigooni D., Rabinovici G.D. (2022). The Role of Amyloid PET in Imaging Neurodegenerative Disorders: A Review. J. Nucl. Med..

[9] Knopman D.S., Amieva H., Petersen R.C., Chételat G., Holtzman D.M., Hyman B.T., Nixon R.A., Jones D.T. (2021). Alzheimer Disease. Nat. Rev. Dis. Prim..

[10] Rajan K.B., Wilson R.S., Weuve J., Barnes L.L., Evans D.A. (2015). Cognitive Impairment 18 Years before Clinical Diagnosis of Alzheimer Disease Dementia. Neurology.

[11] (2020). 2020 Alzheimer’s Disease Facts and Figures. Alzheimers Dement..

[12] Long J.M., Holtzman D.M. (2019). Alzheimer Disease: An Update on Pathobiology and Treatment Strategies. Cell.

[13] Marasco R.A. (2020). Current and Evolving Treatment Strategies for the Alzheimer Disease Continuum. Am. J. Manag. Care.

[14] Shi J., Sabbagh M.N., Vellas B. (2020). Alzheimer’s Disease beyond Amyloid: Strategies for Future Therapeutic Interventions. BMJ.

[15] (2021). 2021 Alzheimer’s Disease Facts and Figures. Alzheimer’s Dement..

[16] Fillit H., Knopman D., Cummings J., Appel F., Schwartz H.L. (1999). Opportunities for Improving Managed Care for Individuals with Dementia: Part 1-The Issues From The Institute for the Study of Aging. Am. J. Manag. Care.

[17] Maciejewska K., Czarnecka K., Szymański P. (2021). A Review of the Mechanisms Underlying Selected Comorbidities in Alzheimer’s Disease. Pharmacol. Rep..

[18] Song C., Shi J., Zhang P., Zhang Y., Xu J., Zhao L., Zhang R., Wang H., Chen H. (2022). Immunotherapy for Alzheimer’s Disease: Targeting β-Amyloid and Beyond. Transl. Neurodegener..

[19] van Dyck C.H. (2018). Anti-Amyloid-β Monoclonal Antibodies for Alzheimer’s Disease: Pitfalls and Promise. Biol. Psychiatry.

[20] Miller C.J., Bioi, Mackinnon R., Yellen G., Timpe L.C. (1991). Segregation of a Missense Mutation in the Amyloid Precursor Protein Gene with Familial Alzheimer’s Disease. Lett. Nat..

[21] Murphy M.P., Levine H. (2010). Alzheimer’s Disease and the Amyloid-β Peptide. J. Alzheimer’s Dis..

[22] Zhang Y.W., Thompson R., Zhang H., Xu H. (2011). APP Processing in Alzheimer’s Disease. Mol. Brain.

[23] Jack C.R., Bennett D.A., Blennow K., Carrillo M.C., Dunn B., Haeberlein S.B., Holtzman D.M., Jagust W., Jessen F., Karlawish J. (2018). NIA-AA Research Framework: Toward a Biological Definition of Alzheimer’s Disease. Alzheimer’s Dement..

[24] Frozza R.L., Lourenco M.V., de Felice F.G. (2018). Challenges for Alzheimer’s Disease Therapy: Insights from Novel Mechanisms beyond Memory Defects. Front. Neurosci..

[25] Patterson C., Feightner J.W., Garcia A., Hsiung G.Y.R., MacKnight C., Sadovnick A.D. (2008). Diagnosis and Treatment of Dementia: 1. Risk Assessment and Primary Prevention of Alzheimer Disease. CMAJ Can. Med. Assoc. J..

[26] Hampel H., Hardy J., Blennow K., Chen C., Perry G., Kim S.H., Villemagne V.L., Aisen P., Vendruscolo M., Iwatsubo T. (2021). The Amyloid-β Pathway in Alzheimer’s Disease. Mol. Psychiatry.

[27] di Fede G., Catania M., Morbin M., Rossi G., Suardi S., Mazzoleni G., Merlin M., Giovagnoli A.R., Prioni S., Erbetta A. (2009). A Recessive Mutation in the APP Gene with Dominant-Negative Effect on Amyloidogenesis. Science.

[28] Hampel H., Vassar R., de Strooper B., Hardy J., Willem M., Singh N., Zhou J., Yan R., Vanmechelen E., de Vos A. (2021). The β-Secretase BACE1 in Alzheimer’s Disease. Biol. Psychiatry.

[29] Brunkan A.L., Goate A.M. (2005). Presenilin Function and γ-Secretase Activity. J. Neurochem..

[30] Zhao J., Liu X., Xia W., Zhang Y., Wang C. (2020). Targeting Amyloidogenic Processing of APP in Alzheimer’s Disease. Front. Mol. Neurosci..

[31] Burdick D., Soreghan B., Kwon M., Kosmoski J., Knauer M., Henschen A., Yatest J., Cotmans C., Glabell C. (1992). Assembly and Aggregation Properties of Synthetic Alzheimer’s A4/β Amyloid Peptide Analogs. J. Biol. Chem..

[32] Ashe K.H., Aguzzi A. (2013). Prions, Prionoids and Pathogenic Proteins in Alzheimer Disease. Prion.

[33] Nelson P.T., Alafuzoff I., Bigio E.H., Bouras C., Braak H., Cairns N.J., Castellani R.J., Crain B.J., Davies P., del Tredici K. (2012). Correlation of Alzheimer Disease Neuropathologic Changes with Cognitive Status: A Review of the Literature. J. Neuropathol. Exp. Neurol..

[34] Hefti F., Goure W.F., Jerecic J., Iverson K.S., Walicke P.A., Krafft G.A. (2013). The Case for Soluble Aβ Oligomers as a Drug Target in Alzheimer’s Disease. Trends Pharmacol. Sci..

[35] Holtzman D.M., Mandelkow E., Selkoe D.J. (2012). Alzheimer Disease in 2020. Cold Spring Harb. Perspect. Med..

[36] Tiwari S., Atluri V., Kaushik A., Yndart A., Nair M. (2019). Alzheimer’s Disease: Pathogenesis, Diagnostics, and Therapeutics. Int. J. Nanomed..

[37] Tarawneh R., Holtzman D.M. (2012). The Clinical Problem of Symptomatic Alzheimer Disease and Mild Cognitive Impairment. Cold Spring Harb. Perspect. Med..

[38] Dubois B., Hampel H., Feldman H.H., Scheltens P., Aisen P., Andrieu S., Bakardjian H., Benali H., Bertram L., Blennow K. (2016). Preclinical Alzheimer’s Disease: Definition, Natural History, and Diagnostic Criteria. Alzheimers Dement..

[39] Lopez O.L., Dekosky S.T. (2018). Clinical Symptoms in Alzheimer’s Disease. Handbook of Clinical Neurology.

[40] Wattmo C., Minthon L., Wallin Å.K. (2016). Mild versus Moderate Stages of Alzheimer’s Disease: Three-Year Outcomes in a Routine Clinical Setting of Cholinesterase Inhibitor Therapy. Alzheimers Res. Ther..

[41] Kumar A., Sidhu J., Goyal A., Tsao J. (2022). Alzheimer Disease.

[42] Apostolova L.G. (2016). Alzheimer Disease. Continuum.

[43] Fu L., Zhang Y., Zhang X., Tian W., Zhang W., Jia Y., Zhang L. (2020). Preparation and in Vitro Activity of Single Chain Antibodies against Alzheimer’s Disease. Immunol. Lett..

[44] Brody D.L., Holtzman D.M. (2008). Active and Passive Immunotherapy for Neurodegenerative Disorders. Annu. Rev. Neurosci..

[45] Gilman S., Koller M., Black R.S., Jenkins L., Griffith S.G., Fox N.C., Eisner L., Kirby L., Rovira M.B., Forette F. (2005). Clinical Effects of A Immunization (AN1792) in Patients with AD in an Interrupted Trial. Neurology.

[46] Lemere C.A. (2013). Immunotherapy for Alzheimer’s Disease: Hoops and Hurdles. Mol. Neurodegener..

[47] Cummings J., Lee G., Nahed P., Kambar M.E.Z.N., Zhong K., Fonseca J., Taghva K. (2022). Alzheimer’s Disease Drug Development Pipeline: 2022. Alzheimers Dement. Transl. Res. Clin. Interv..

[48] Pardridge W.M. (2020). Treatment of Alzheimer’s Disease and Blood–Brain Barrier Drug Delivery. Pharmaceuticals.

[49] Uhlmann R.E., Rother C., Rasmussen J., Schelle J., Bergmann C., Ullrich Gavilanes E.M., Fritschi S.K., Buehler A., Baumann F., Skodras A. (2020). Acute Targeting of Pre-Amyloid Seeds in Transgenic Mice Reduces Alzheimer-like Pathology Later in Life. Nat. Neurosci..

[50] Banks W.A. (2016). From Blood-Brain Barrier to Blood-Brain Interface: New Opportunities for CNS Drug Delivery. Nat. Rev. Drug. Discov..

[51] Bard F., Cannon C., Barbour R., Burke R.L., Games D., Grajeda H., Guido T., Hu K., Huang J., Johnson-Wood K. (2000). Peripherally Administered Antibodies against Amyloid β-Peptide Enter the Central Nervous System and Reduce Pathology in a Mouse Model of Alzheimer Disease. Nat. Med..

[52] Adolfsson O., Pihlgren M., Toni N., Varisco Y., Buccarello A.L., Antoniello K., Lohmann S., Piorkowska K., Gafner V., Atwal J.K. (2012). An Effector-Reduced Anti-β-Amyloid (Aβ) Antibody with Unique Aβ Binding Properties Promotes Neuroprotection and Glial Engulfment of Aβ. J. Neurosci..

[53] Solomon B., Koppel R., Hanan E., Katzav T. (1996). Monoclonal Antibodies Inhibit in Vitro Fibrillar Aggregation of the Alzheimer B-Amyloid Peptide. Proc. Natl. Acad. Sci. USA.

[54] Shi M., Chu F., Zhu F., Zhu J. (2022). Impact of Anti-Amyloid-β Monoclonal Antibodies on the Pathology and Clinical Profile of Alzheimer’s Disease: A Focus on Aducanumab and Lecanemab. Front. Aging Neurosci..

[55] Dunn B., Stein P., Cavazzoni P. (2021). Approval of Aducanumab for Alzheimer Disease—The FDA’s Perspective. JAMA Intern. Med..

[56] Ebell M.H., Barry H.C. (2022). Why Physicians Should Not Prescribe for Alzheimer Disease. Am. Fam. Physician.

[57] Bohrmann B., Baumann K., Benz J., Gerber F., Huber W., Knoflach F., Messer J., Oroszlan K., Rauchenberger R., Richter W.F. (2012). Gantenerumab: A Novel Human Anti-Aβ Antibody Demonstrates Sustained Cerebral Amyloid-β Binding and Elicits Cell-Mediated Removal of Human Amyloid-β. J. Alzheimers Dis..

[58] (2022). Media & Investor Release Ad Hoc Announcement Pursuant to Art. 53 LR Roche Provides Update on Phase III GRADUATE Programme Evaluating Gantenerumab in Early Alzheimer’s Disease.

[59] Salloway S., Honigberg L.A., Cho W., Ward M., Friesenhahn M., Brunstein F., Quartino A., Clayton D., Mortensen D., Bittner T. (2018). Amyloid Positron Emission Tomography and Cerebrospinal Fluid Results from a Crenezumab Anti-Amyloid-β Antibody Double-Blind, Placebo-Controlled, Randomized Phase II Study in Mild-to-Moderate Alzheimer’s Disease (BLAZE). Alzheimers Res. Ther..

[60] Ostrowitzki S., Bittner T., Sink K.M., Mackey H., Rabe C., Honig L.S., Cassetta E., Woodward M., Boada M., van Dyck C.H. (2022). Evaluating the Safety and Efficacy of Crenezumab vs Placebo in Adults with Early Alzheimer Disease: Two Phase 3 Randomized Placebo-Controlled Trials. JAMA Neurol..

[61] Hardy J., Bogdanovic N., Winblad B., Portelius E., Andreasen N., Cedazo-Minguez A., Zetterberg H. (2014). Pathways to Alzheimer’s Disease. J. Intern. Med..

[62] Doody R.S., Thomas R.G., Farlow M., Iwatsubo T., Vellas B., Joffe S., Kieburtz K., Raman R., Sun X., Aisen P.S. (2014). Phase 3 Trials of Solanezumab for Mild-to-Moderate Alzheimer’s Disease. N. Engl. J. Med..

[63] DeMattos R.B., Lu J., Tang Y., Racke M.M., DeLong C.A., Tzaferis J.A., Hole J.T., Forster B.M., McDonnell P.C., Liu F. (2012). A Plaque-Specific Antibody Clears Existing β-Amyloid Plaques in Alzheimer’s Disease Mice. Neuron.

[64] Bouter Y., Liekefeld H., Pichlo S., Westhoff A.C., Fenn L., Bakrania P., Bayer T.A. (2022). Donanemab Detects a Minor Fraction of Amyloid-β Plaques in Post-Mortem Brain Tissue of Patients with Alzheimer’s Disease and Down Syndrome. Acta Neuropathol..

[65] Mintun M., Lo A., Evans C., Wessels A., Ardayfio P., Andersen S., Shcherbinin S., Sparks J., Sims J., Brys M. (2021). Donanemab in Early Alzheimer’s Disease. N. Engl. J. Med..

[66] Olloquequi J., Ettcheto M., Cano A., Sanchez-López E., Carrasco M., Espinosa T., Beas-Zarate C., Gudiño-Cabrera G., Ureña-Guerrero M.E., Verdaguer E. (2022). Impact of New Drugs for Therapeutic Intervention in Alzheimer’s Disease. Front. Biosci. Landmark.

[67] Parrocha C.M.T., Nowick J.S. (2023). Current Peptide Vaccine and Immunotherapy Approaches against Alzheimer’s Disease. Pept. Sci..

[68] Lecanemab Confirmatory Phase 3 Clarity Ad Study Met Primary Endpoint, Showing Highly Statistically Significant Reduction of Clinical Decline in Large Global Clinical Study of 1795 Participants with Early Alzheimer’s Disease. https://investors.biogen.com/news-releases/news-release-details/lecanemab-confirmatory-phase-3-clarity-ad-study-met-primary.

[69] vander Zanden C.M., Chi E.Y. (2020). Passive Immunotherapies Targeting Amyloid β and Tau Oligomers in Alzheimer’s Disease. J. Pharm. Sci..

[70] Tucker S., Möller C., Tegerstedt K., Lord A., Laudon H., Sjödahl J., Söderberg L., Spens E., Sahlin C., Waara E.R. (2015). The Murine Version of BAN2401 (MAb158) Selectively Reduces Amyloid-β Protofibrils in Brain and Cerebrospinal Fluid of Tg-ArcSwe Mice. J. Alzheimers Dis..

[71] Söllvander S., Nikitidou E., Gallasch L., Zyśk M., Söderberg L., Sehlin D., Lannfelt L., Erlandsson A. (2018). The Aβ Protofibril Selective Antibody MAb158 Prevents Accumulation of Aβ in Astrocytes and Rescues Neurons from Aβ-Induced Cell Death. J. Neuroinflamm..

[72] Swanson C.J., Zhang Y., Dhadda S., Wang J., Kaplow J., Lai R.Y.K., Lannfelt L., Bradley H., Rabe M., Koyama A. (2021). A Randomized, Double-Blind, Phase 2b Proof-of-Concept Clinical Trial in Early Alzheimer’s Disease with Lecanemab, an Anti-Aβ Protofibril Antibody. Alzheimers Res. Ther..

[73] van Dyck C.H., Swanson C.J., Aisen P., Bateman R.J., Chen C., Gee M., Kanekiyo M., Li D., Reyderman L., Cohen S. (2022). Lecanemab in Early Alzheimer’s Disease. N. Engl. J. Med..

[74] FDA Grants Accelerated Approval for Alzheimer’s Disease Treatment. https://www.fda.gov/news-events/press-announcements/fda-grants-accelerated-approval-alzheimers-disease-treatment.

[75] Armstrong A. Facing a Familiar Side Effect Problem, Eisai Makes the Case for Its next Alzheimer’s Drug after Patient Deaths. https://www.fiercebiotech.com/biotech/eisai-biogen-lecanemab-alzheimers-aria-patient-deaths-adverse-events.

[76] Panza F., Lozupone M., Logroscino G., Imbimbo B.P. (2019). A Critical Appraisal of Amyloid-β-Targeting Therapies for Alzheimer Disease. Nat. Rev. Neurol..

[77] Illouz T., Madar R., Hirsh T., Biragyn A., Okun E. (2021). Induction of an Effective Anti-Amyloid-β Humoral Response in Aged Mice. Vaccine.

[78] Davtyan H., Ghochikyan A., Petrushina I., Hovakimyan A., Davtyan A., Poghosyan A., Marleau A.M., Movsesyan N., Kiyatkin A., Rasool S. (2013). Immunogenicity, Efficacy, Safety, and Mechanism of Action of Epitope Vaccine (Lu AF20513) for Alzheimer’s Disease: Prelude to a Clinical Trial. J. Neurosci..

[79] Orgogozo J.-M., Gilman S., Dartigues J.-F., Laurent B., Puel M., Kirby L.C., Jouanny P., Dubois B., Eisner L., Flitman S. (2003). Subacute Meningoencephalitis in a Subset of Patients with AD after A42 Immunization. Neurology.

[80] Maier M., Seabrook T.J., Lazo N.D., Jiang L., Das P., Janus C., Lemere C.A. (2006). Short Amyloid-β (Aβ) Immunogens Reduce Cerebral Aβ Load and Learning Deficits in an Alzheimer’s Disease Mouse Model in the Absence of an Aβ-Specific Cellular Immune Response. J. Neurosci..

[81] Wiessner C., Wiederhold K.H., Tissot A.C., Frey P., Danner S., Jacobson L.H., Jennings G.T., Lüönd R., Ortmann R., Reichwald J. (2011). The Second-Generation Active Aβ Immunotherapy CAD106 Reduces Amyloid Accumulation in APP Transgenic Mice While Minimizing Potential Side Effects. J. Neurosci..

[82] Lambracht-Washington D., Rosenberg R.N. (2013). Advances in the Development of Vaccines for Alzheimer’s Disease. Discov. Med..

[83] Bachmann M.F., Jennings G.T., Vogel M. (2019). A Vaccine against Alzheimer`s Disease: Anything Left but Faith?. Expert. Opin. Biol. Ther..

[84] Winblad B., Andreasen N., Minthon L., Floesser A., Imbert G., Dumortier T., Maguire P., Blennow K., Lundmark J., Staufenbiel M. (2012). Safety, Tolerability, and Antibody Response of Active Abeta Immunotherapy with CAD106 in Patients with Alzheimer’s Disease: Randomised, Double-Blind, Placebo-Controlled, First-in-Human Study. LancetNeurol.

[85] Vandenberghe R., Riviere M.E., Caputo A., Sovago J., Maguire R.P., Farlow M., Marotta G., Sanchez-Valle R., Scheltens P., Ryan J.M. (2017). Active Aβ Immunotherapy CAD106 in Alzheimer’s Disease: A Phase 2b Study. Alzheimers Dement. Transl. Res. Clin. Interv..

[86] Riviere M., Turner R.S., Sui Y., Laurent N., Langbaum J.B., Cazorla P., Ricart J., Seneca N., Caputo A., Reiman E.M. (2021). API Generation Program: Active Immunotherapy CAD106 Slows Amyloid Deposition in Cognitively Unimpaired APOE4 Homozygotes. Alzheimers Dement..

[87] Huang X. (2020). Alzheimer’s Disease: Drug Discovery.

[88] Lacosta A.M., Pascual-Lucas M., Pesini P., Casabona D., Pérez-Grijalba V., Marcos-Campos I., Sarasa L., Canudas J., Badi H., Monleón I. (2018). Safety, Tolerability and Immunogenicity of an Active Anti-Aβ 40 Vaccine (ABvac40) in Patients with Alzheimer’s Disease: A Randomised, Double-Blind, Placebo-Controlled, Phase i Trial. Alzheimers Res. Ther..

[89] Fettelschoss A., Zabel F., Bachmann M.F. (2014). Vaccination against Alzheimer Disease: An Update on Future Strategies. Hum. Vaccin. Immunother..

[90] Cacabelos R. (2020). How Plausible Is an Alzheimer’s Disease Vaccine?. Expert Opin. Drug Discov..

[91] Pike C.J. (2017). Sex and the Development of Alzheimer’s Disease. J. Neurosci. Res..

[92] Armstrong R.A. (2019). Risk Factors for Alzheimer’s Disease. Folia. Neuropathol..

[93] Mendez M.F. (2017). Early-Onset Alzheimer Disease. Neurol. Clin..

[94] Ballard C., Gauthier S., Corbett A., Brayne C., Aarsland D., Jones E. (2011). Alzheimer’s Disease. Lancet.

[95] van Cauwenberghe C., van Broeckhoven C., Sleegers K. (2016). The Genetic Landscape of Alzheimer Disease: Clinical Implications and Perspectives. Genet. Med..

[96] Li N.M., Liu K.F., Qiu Y.J., Zhang H.H., Nakanishi H., Qing H. (2019). Mutations of β-Amyloid Precursor Protein Alter the Consequence of Alzheimer’s Disease Pathogenesis. Neural Regen. Res..

[97] de Strooper B. (2007). Loss-of-Function Presenilin Mutations in Alzheimer Disease. Talking Point on the Role of Presenilin Mutations in Alzheimer Disease. EMBO Rep..

[98] Silva M.V.F., Loures C.D.M.G., Alves L.C.V., de Souza L.C., Borges K.B.G., Carvalho M.D.G. (2019). Alzheimer’s Disease: Risk Factors and Potentially Protective Measures. J. Biomed. Sci..

[99] Cahill L. (2006). Why Sex Matters for Neuroscience. Nat. Rev. Neurosci..

[100] Breijyeh Z., Karaman R. (2020). Comprehensive Review on Alzheimer’s Disease: Causes and Treatment. Molecules.

[101] Buckley R.F., Mormino E.C., Amariglio R.E., Properzi M.J., Rabin J.S., Lim Y.Y., Papp K.V., Jacobs H.I.L., Burnham S., Hanseeuw B.J. (2018). Sex, Amyloid, and APOE Ε4 and Risk of Cognitive Decline in Preclinical Alzheimer’s Disease: Findings from Three Well-Characterized Cohorts. Alzheimers Dement..

[102] Li X., Song D., Leng S.X. (2015). Link between Type 2 Diabetes and Alzheimer’s Disease: From Epidemiology to Mechanism and Treatment. Clin. Interv. Aging.

[103] Livingston G., Huntley J., Sommerlad A., Ames D., Ballard C., Banerjee S., Brayne C., Burns A., Cohen-Mansfield J., Cooper C. (2020). Dementia Prevention, Intervention, and Care: 2020 Report of the Lancet Commission. Lancet.

[104] Uwishema O., Mahmoud A., Sun J., Correia I.F.S., Bejjani N., Alwan M., Nicholas A., Oluyemisi A., Dost B. (2022). Is Alzheimer’s Disease an Infectious Neurological Disease? A Review of the Literature. Brain Behav..

[105] Vaz M., Silvestre S. (2020). Alzheimer’s Disease: Recent Treatment Strategies. Eur. J. Pharmacol..

[106] (2019). 2019 Alzheimer’s Disease Facts and Figures. Alzheimers Dement..

[107] Lemere C.A., Masliah E. (2010). Can Alzheimer Disease Be Prevented by Amyloid-Β Immunotherapy?. Nat. Rev. Neurol..

